# Supplementary material for: Deciphering polymorphism in 61,157 Escherichia coli genomes via epistatic sequence landscapes
Source: Nat Commun. 2022 Jul 12;13:4030. doi: 10.1038/s41467-022-31643-3 (PMC9276797; doi:10.1038/s41467-022-31643-3)
Supplement: Supplementary file 1 — Supplementary Information [file 41467_2022_31643_MOESM1_ESM.pdf]

Deciphering polymorphism in 61,157 *Escherichia coli* genomes via epistatic sequence landscapes

Supplementary Information

Contents

Supplementary Notes **3**
Controlling for phylogenetic bias in DCA . . . 3

Supplementary Methods **4**
MSA randomization procedure to control for phylogenetic couplings . . . 4

Supplementary Figures **5**
Supplementary Figure 1 . . . 5
Supplementary Figure 2 . . . 6
Supplementary Figure 3 . . . 8
Supplementary Figure 4 . . . 9
Supplementary Figure 5 . . . 10
Supplementary Figure 6 . . . 11
Supplementary Figure 7 . . . 12
Supplementary Figure 8 . . . 13
Supplementary Figure 9 . . . 14
Supplementary Figure 10 . . . 15

Supplementary References **16**

Supplementary Notes

Controlling for phylogenetic bias in DCA

DCA assumes that amino-acid sequences observed in nature represent an (up to simple reweighting) independent sample from an unknown probability distribution. Phylogenetic relationships between members of the same protein family necessarily violate this hypothesis. To date there is no known method to incorporate phylogenetic relations into coevolutionary modeling of amino-acid sequences, but to assess the influence of phylogeny one can follow the methodology developed by Horta and Weigt [1]. Instead of trying to disentangle functional couplings from phylogenetic ones, they have proposed null models reproducing conservation and phylogenetic patterns observed in the original MSA, but removing any signature of coevolution between residues. Comparing results obtained from DCA models trained on true MSAs to those obtained with DCA models trained on corresponding randomized MSAs, we can assess whether "epistatic" signals detected by DCA are actually caused by phylogenetic correlations.

Here, we use null model II [1]: it randomizes MSA to have both similar position-specific frequencies of amino acids and similar pairwise Hamming distances between sequences (Supplementary Methods section *MSA randomization procedure to control for phylogenetic couplings*). The former ensures the conservation of the MSA profile, meaning that an IND model will give equivalent results if trained on the randomized MSA compared to the original one. The conservation of the pairwise distances between MSA sequences allows to reproduce phylogenetic patterns contained in the original MSA. On the contrary, no coevolutionary signal is contained in the randomized MSA, in difference to the original one. Due to the high computational cost of this randomization procedure, we have chosen to run it on a subset of 51 Pfam MSAs randomly selected among the 2,053 screened in our work. We observe that a DCA model trained on a randomized MSA generates a CDE which takes intermediate values between those of the original CDE and CIE (Supplementary Figure 10a). This reduction in entropy compared to the entropy of an independent model is an expected outcome of the addition of phylogenetic couplings into the DCA modeling procedure. However, we note that phylogenetic couplings are not sufficient to explain the very low CDE observed in the original data. This indicates that structural and functional coevolution play an essential role in constraining CDE values.

In our work, we have used CDE to predict polymorphic and conserved sites in *E. coli.* We have taken care of excluding any sequence with more than 90% identity with *E. coli* reference strain. Given that the average variability within *E. coli* species only reaches 2%, DCA predictions of polymorphisms give a way to assess its out-of-sample performance. The patterns of conservation and variability within *E. coli* are the results of selection, not phylogeny. This is illustrated by the lower performance of DCA models trained on randomized MSAs in predicting polymorphisms compared to the predictions made by IND models and by DCA models trained on original MSAs (Supplementary Figure 10b). In contrast, DCA models trained on original MSAs outperform IND models, meaning that their predictions rely more on structural or functional coevolutionary couplings than on phylogenetic correlations.

Taken altogether, these results suggest that — even if DCA may incorporate phylogenetic couplings — these do not account for the majority of the signals we detect. These findings are in line with conclusions in [1] and DCA's ability to predict functional protein variants [2].

Supplementary Methods

MSA randomization procedure to control for phylogenetic couplings

Among the 2,053 inter-species Pfam MSAs used to train models to study inter-strain data, 51 MSAs were randomly selected. Using published algorithms from Horta and Weigt [1], 51 randomized MSAs were produced with Null model II. The corresponding DCA models were trained on these MSAs and the CDE was computed with the randomized reference sequence taken for context. Independent models and DCA models trained with the corresponding original MSAs were used to compare their performance to those of the DCA models trained on randomized MSAs.

Supplementary Figures

Supplementary Figure 1

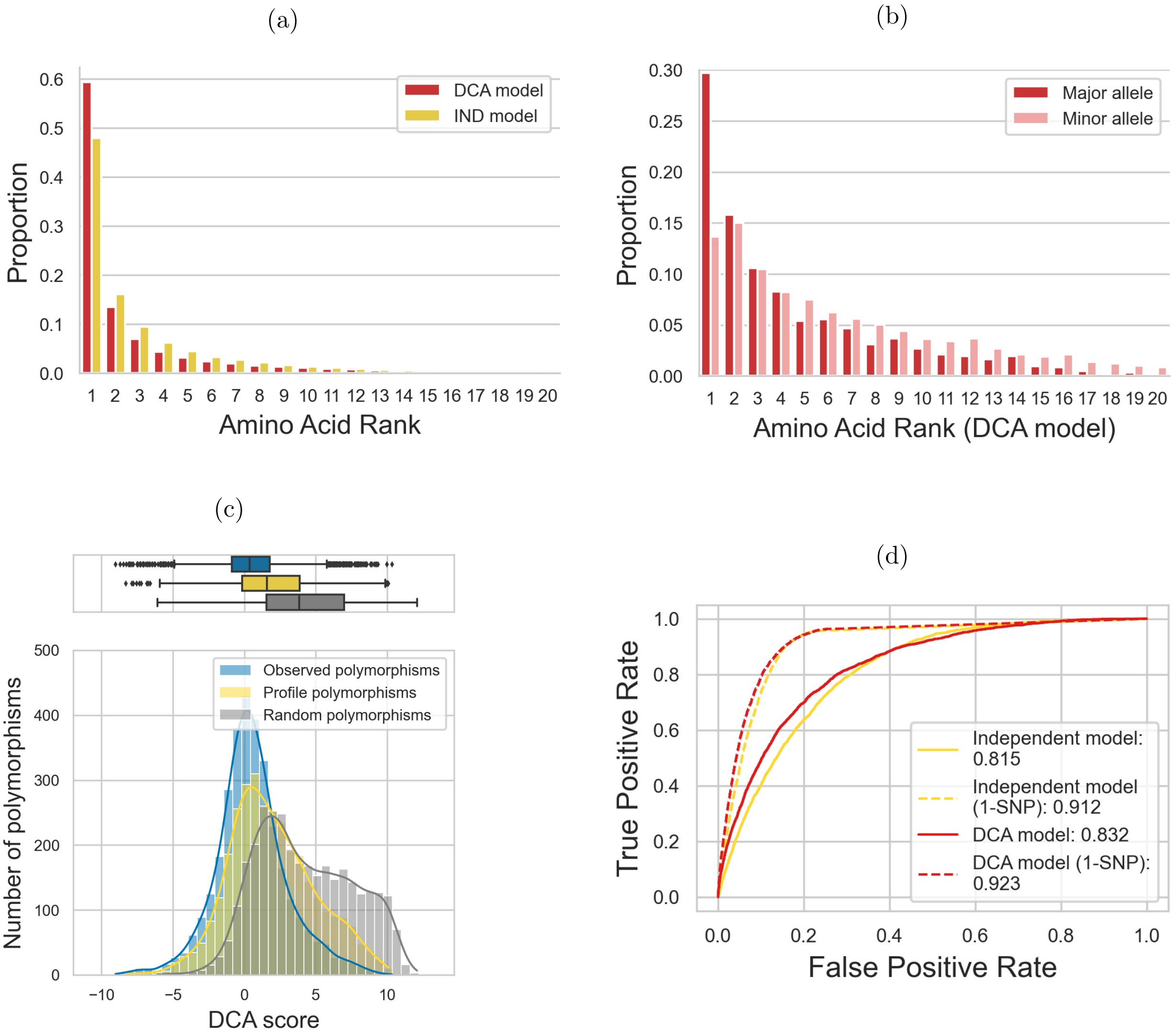

Supplementary Figure 1: **Predicted effects of observed amino acids using an IND model that neglects epistasis or a DCA model that incorporates pairwise epistasis. Models trained on full genes.** **(a)** Rank of native amino acid in the reference strain as compared to all 20 possible amino acids for DCA model (red) and IND model (yellow). **(b)** DCA rank of major (red) and minor (pink) allele for all sites that are polymorphic at a >5%-threshold, among all 20 possible amino acids. **(c)** Distribution of DCA scores of non-synonymous polymorphisms observed at frequencies >5% across the >60,000 strains (blue) compared to mutations sampled from an IND model (yellow) or to random mutations (grey). Boxplot center lines represent medians, box limits are upper and lower quartiles, whiskers extend to show the rest of the distribution within an 1.5× interquartile range, outliers are represented with points; sample size is 3,253 mutations for each of the three groups. **(d)** ROC curves of different models for predicting polymorphisms observed at >5% frequency in *E. coli* (DCA models in red, IND models in yellow, solid lines correspond to models where all possible mutations at a site are considered, dashed lines correspond to models that are restricted to 1-SNP mutations).

Supplementary Figure 2

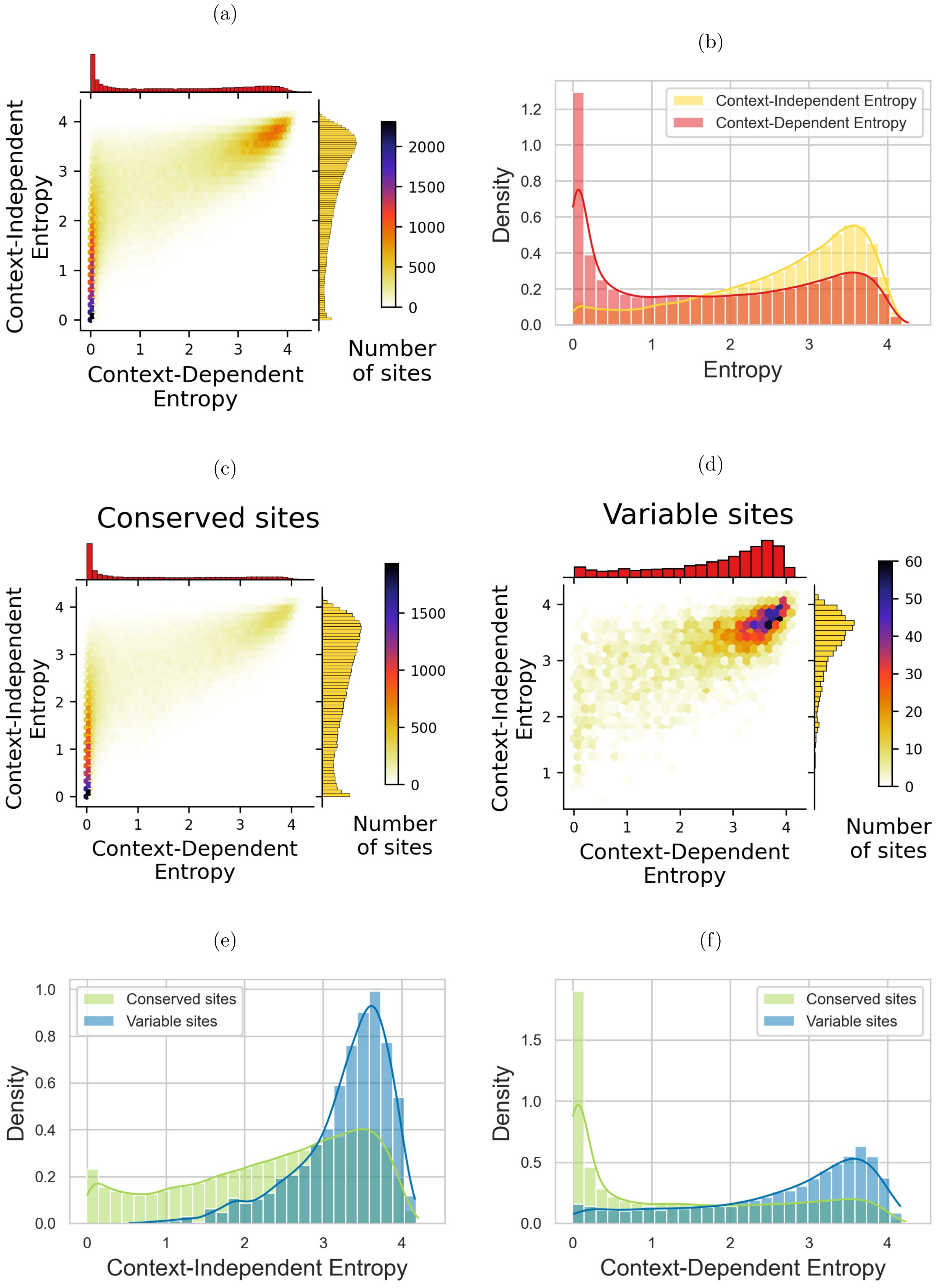

(g) (h)

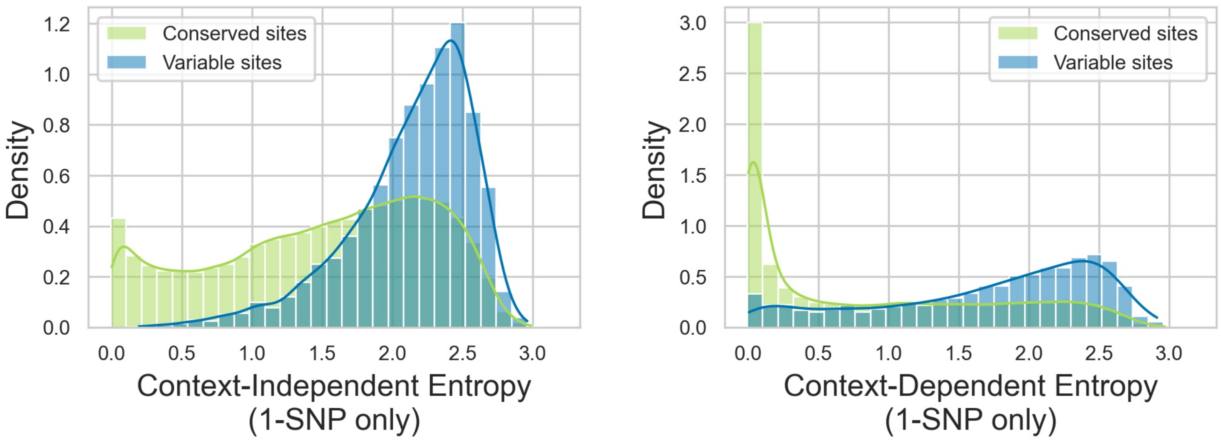

Supplementary Figure 2: **Predicting the variability of amino-acid sites and amino-acid sites that are conserved or polymorphic in *E. coli*. Comparison of the performance of an IND and a DCA models trained on full genes.** **(a)** Bivariate histogram of CDE and CIE for all sites in the dataset. **(b)** Marginal distributions of CDE (red) and CIE (yellow) for all sites in the dataset. **(c)** Bivariate histogram of CDE and CIE for sites that are conserved across >60,000 strains of *E. coli*. **(d)** Bivariate histogram of CDE and CIE for sites that are polymorphic at a 5% threshold across >60,000 strains of *E. coli*. **(e)** Distribution of CIE for conserved (green) and polymorphic (blue) sites in *E. coli*. **(f)** Distribution of CDE for conserved (green) and polymorphic (blue) sites in *E. coli*. **(g)** Distribution of CIE restricted to 1-SNP mutations for conserved (green) and polymorphic (blue) sites in *E. coli*. **(h)** Distribution of CDE restricted to 1-SNP mutations for conserved (green) and polymorphic (blue) sites in *E. coli*.

Supplementary Figure 3

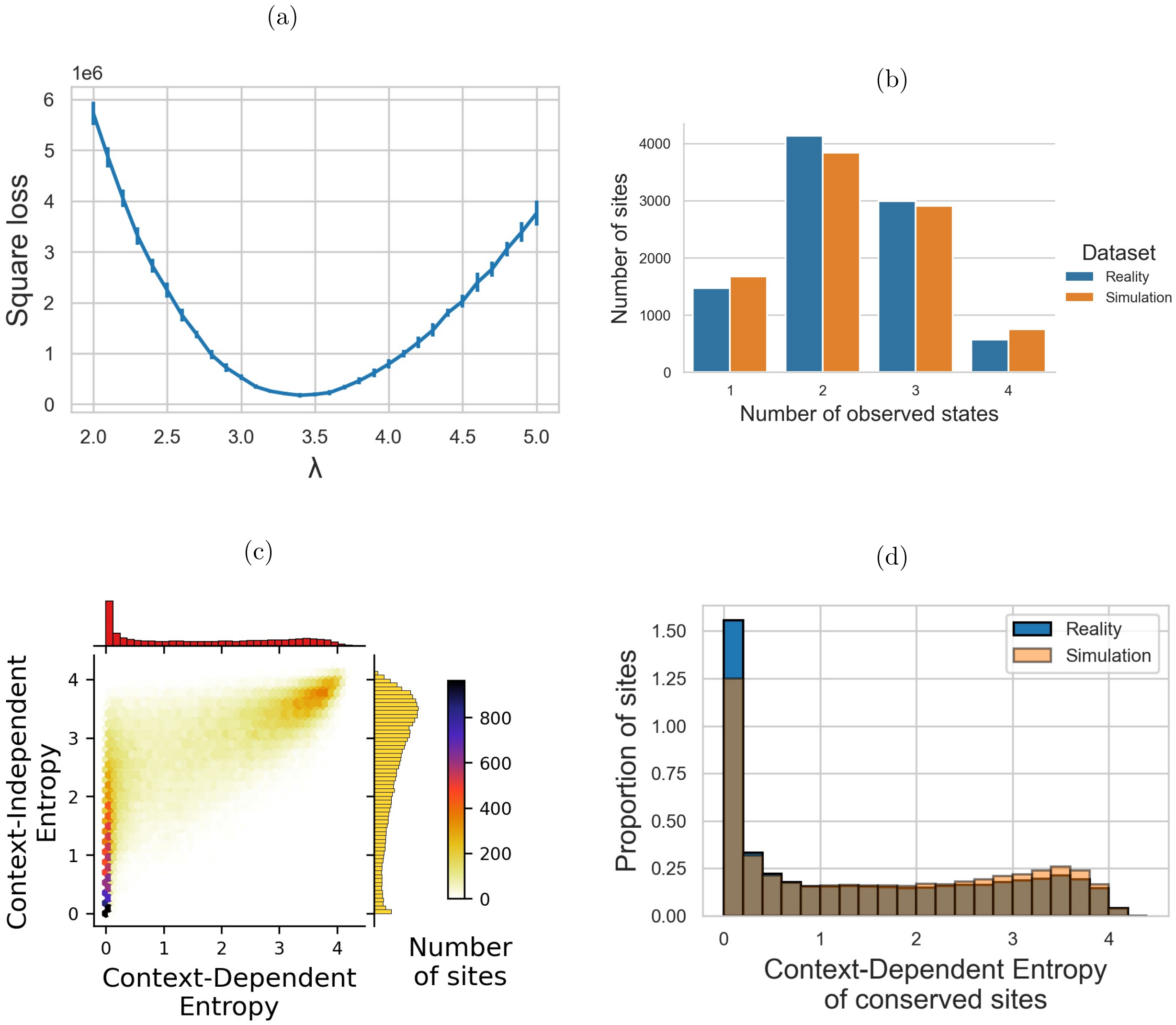

Supplementary Figure 3: **Simulations of synonymous and non-synonymous diversity occuring on full genes (Methods section *Simulations of neutral diversity segregating on amino-acid sites*).** **(a)** Simulation of synonymous diversity. For each λ ranging from 2 to 5 with a 0.1 step-size, 20 simulations are run. The square loss between the amount of simulated synonymous diversity and the real amount observed in the dataset is computed. The best λ parameter is 3.1. Square loss is presented as average value across 20 simulations ± 1 standard deviation. **(b)** Simulation of synonymous diversity. Average results of the 20 simulations of synonymous diversity with $\lambda = 3.1$. We have focussed on sites where there are exactly four possible 1-SNP synonymous mutations. As we can see observed synonymous diversity is not saturated (sites with all four possible synonymous codons observed in the dataset are rare). Simulations (orange) achieve a very good fit of the observed reality (blue) even with a basic model like JC69 that ignores differences in mutation rates between nucleotide pairs. **(c)** Simulation of non-synonymous diversity. Bivariate histogram of CDE and CIE for sites that are conserved in the simulated dataset produced with parameter $\lambda = 3.1$. Most of the sites cluster on the left peak of low CDE. However, as observed in the real dataset, some of the sites where no mutation occurred have a high CDE. **(d)** Simulation of non-synonymous diversity. Comparison of CDE distributions of real conserved sites (sites conserved across >60,000 strains in the dataset, in blue) and simulated conserved sites (sites where no mutation was simulated, in orange).

Supplementary Figure 4

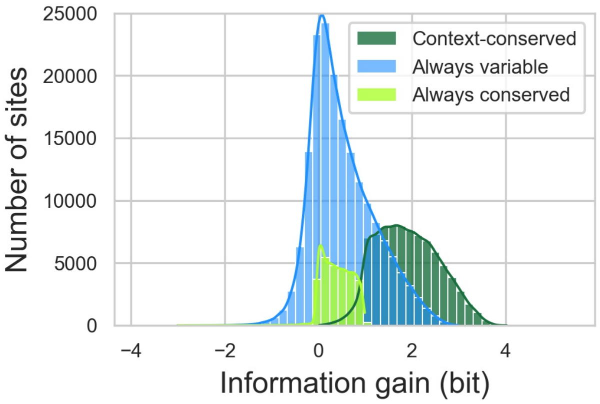

Supplementary Figure 4: **Quantifying the effect of the context in reducing amino-acid site variability with models trained on full genes.** Information gain quantifies the difference between an amino-acid site variability across distant species and its potential variability in *E. coli*. Sites that are variable across distant species (CIE$\geq$1) but conserved in *E. coli* (CDE<1) are the ones with the highest information gains (dark green distribution).

Supplementary Figure 5

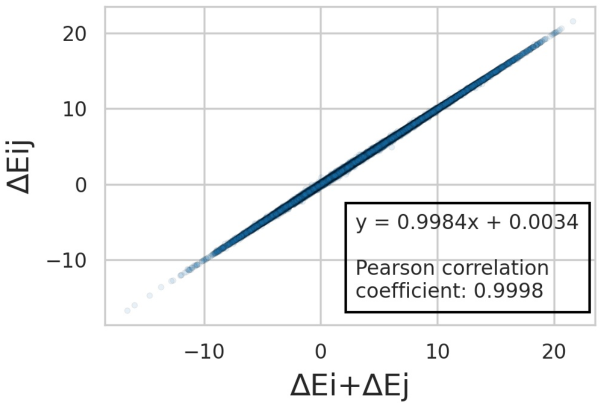

Supplementary Figure 5: **Epistasis observed in *E. coli* with models trained on full genes.** Mutational effect ΔE_{ij} of observed double mutations with respect to the reference, plotted against the sum $\Delta E_i + \Delta E_j$ of the individual mutations.

Supplementary Figure 6

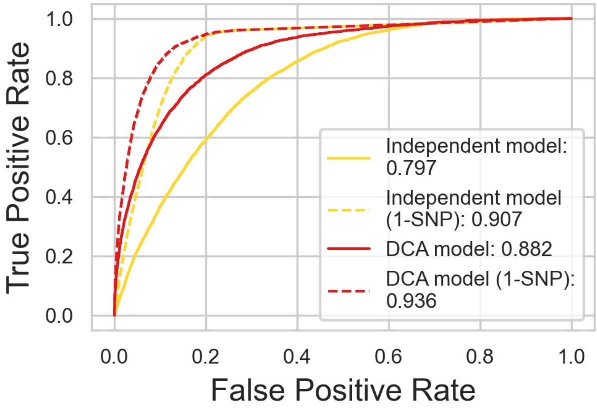

Supplementary Figure 6: **ROC curves of different models for predicting polymorphisms observed at >5% frequency in *E. coli.* Models trained on Pfam domains.** DCA models are in red, IND models in yellow, solid lines correspond to models where all possible mutations at a site are considered, dashed lines correspond to models that are restricted to 1-SNP mutations.

Supplementary Figure 7

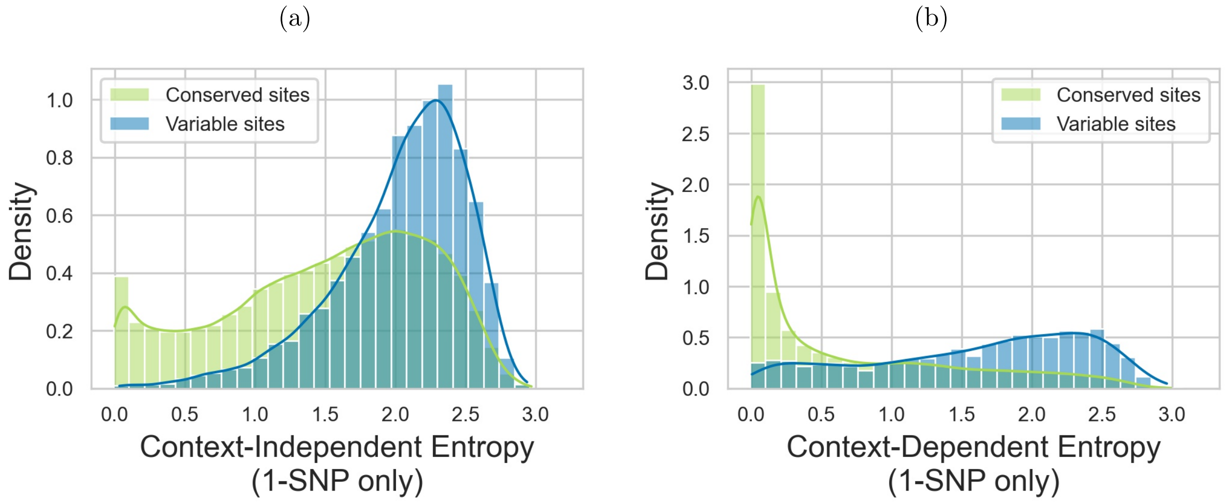

Supplementary Figure 7: **CIE and CDE computed for 1-SNP mutations from the reference codon with models trained on Pfam domains.** The total number of amino acids that can be observed with no more than 1 SNP never exceeds 9, corresponding to a maximal entropy value of about 3.2. **(a)** Distribution of CIE restricted to 1-SNP mutations for conserved (green) and polymorphic (blue) sites in *E. coli*. **(b)** Distribution of CDE restricted to 1-SNP mutations for conserved (green) and polymorphic (blue) sites in *E. coli*.

Supplementary Figure 8

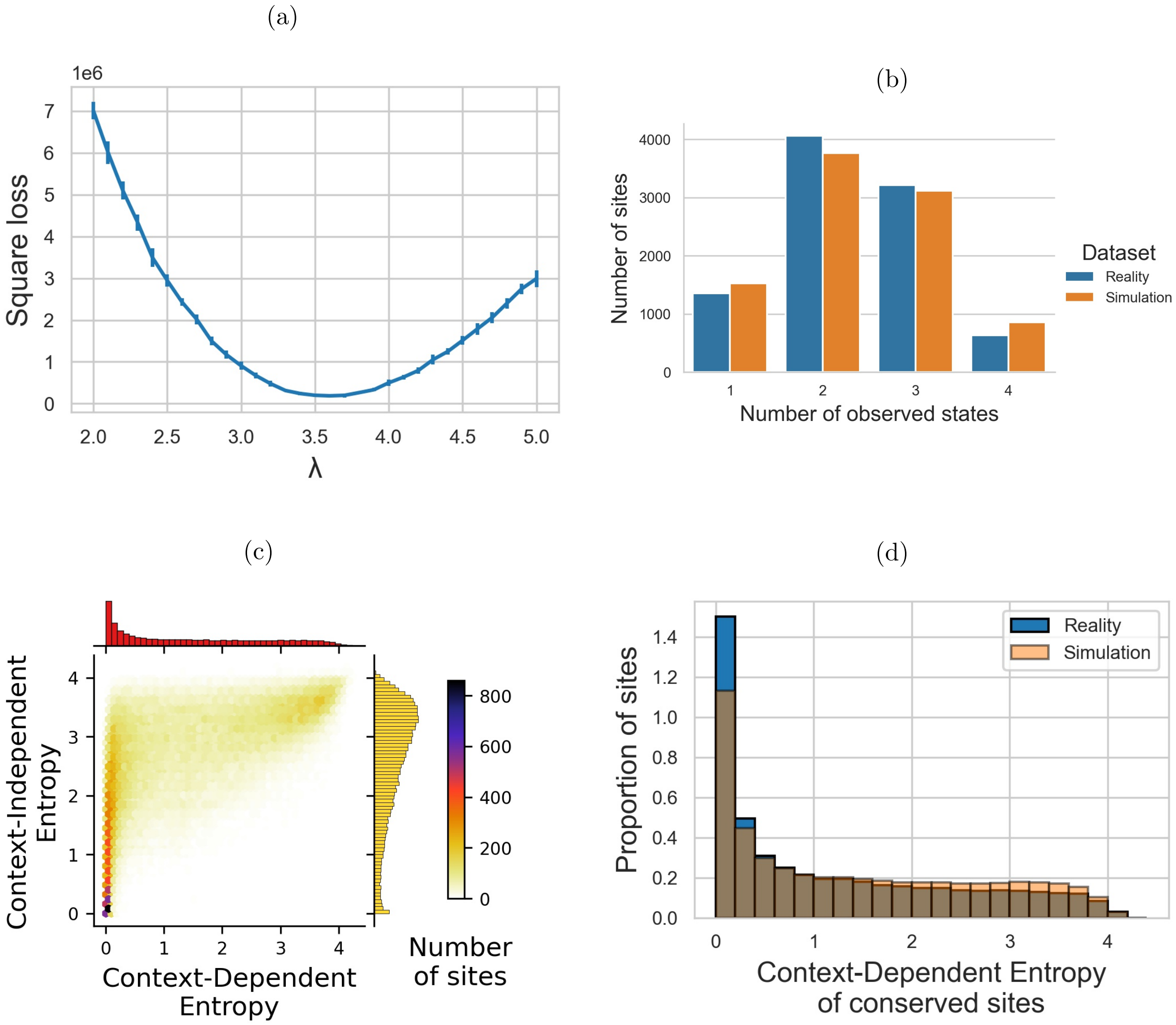

Supplementary Figure 8: **Simulations of synonymous and non-synonymous diversity occuring on Pfam domains (Methods section *Simulations of neutral diversity segregating on amino-acid sites*). (a)** Simulation of synonymous diversity. For each λ ranging from 2 to 5 with a 0.1 step-size, 20 simulations are run. The square loss between the amount of simulated synonymous diversity and the real amount observed in the dataset is computed. The best λ parameter is 3.6. Square loss is presented as average value across 20 simulations ± 1 standard deviation. **(b)** Simulation of synonymous diversity. Average results of the 20 simulations of synonymous diversity with $\lambda = 3.6$. We have focussed on sites where there are exactly four possible 1-SNP synonymous mutations. As we can see synonymous diversity is not saturated (sites with all four possible synonymous codons observed in the dataset are rare). Simulations (in orange) achieve good fit of the observed reality (in blue) even with a basic model like JC69 that ignores differences in mutation rates between nucleotide pairs. **(c)** Simulation of non-synonymous diversity. Bivariate histogram of CDE and CIE for sites that are conserved in the simulated dataset produced with parameter $\lambda = 3.6$. Most of the sites cluster on the left peak of low CDE. However, as observed in the real dataset, some of the sites where no mutation occurred have a high CDE. **(d)** Comparison of CDE distributions of real conserved sites (sites conserved across >60,000 strains in the dataset, in blue) and simulated conserved sites (sites where no mutation was simulated, in orange).

Supplementary Figure 9

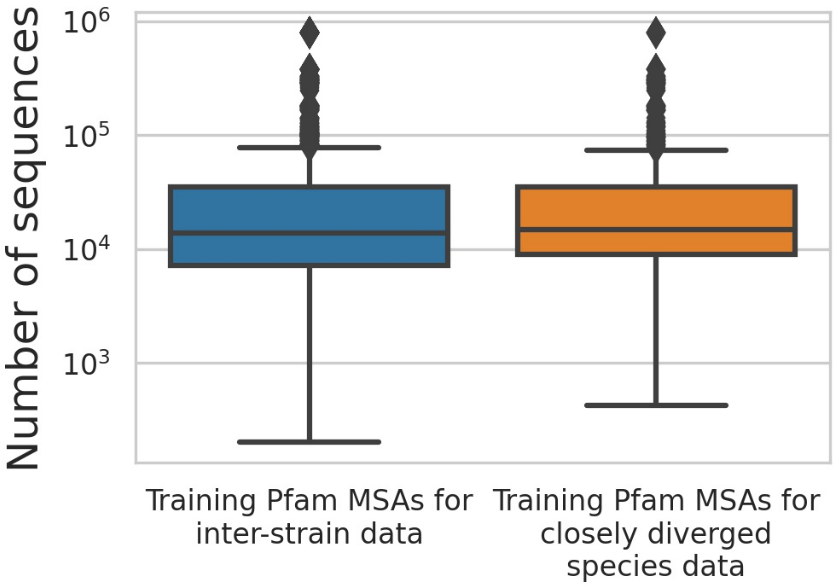

Supplementary Figure 9: **Boxplot of the number of sequences in Pfam inter-species MSAs used to train DCA models.** MSAs used to train DCA models for analysis of inter-strain data (in blue) have a median of 50,988 sequences and a 5th percentile of 913 sequences. MSAs used to train DCA models for analysis of closely diverged species (in orange) data have a median of 41,372 sequences and a 5th percentile of 2,321 sequences. Boxplot center lines represent medians, box limits are upper and lower quartiles, whiskers extend to show the rest of the distribution within an $1.5\times$ interquartile range, outliers are represented with points.

Supplementary Figure 10

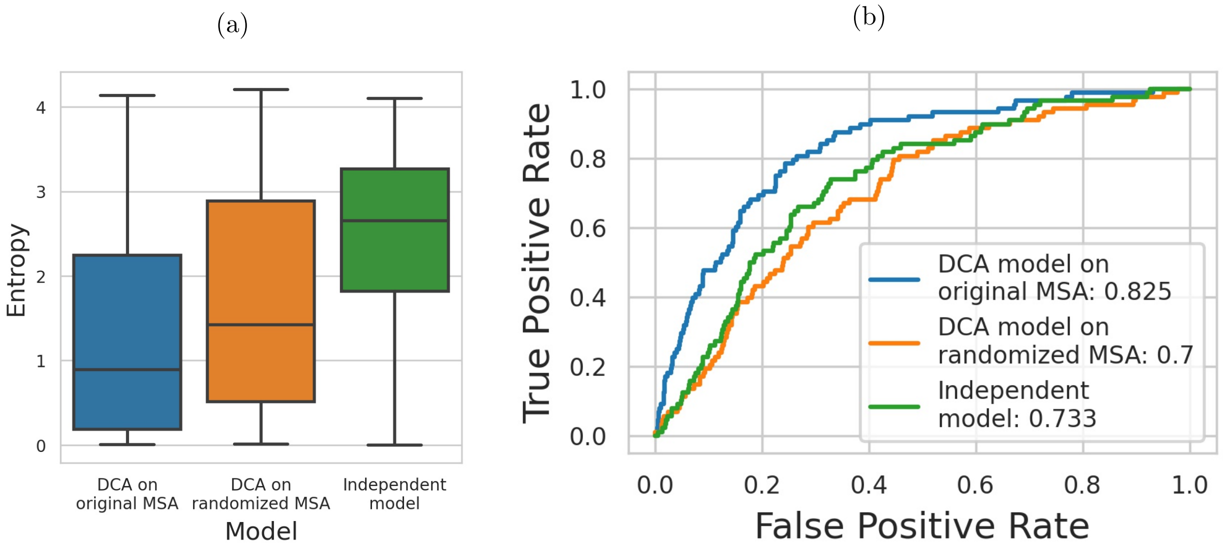

Supplementary Figure 10: **Controlling for phylogenetic biases, comparison of model performance on 51 Pfam domains (blue: DCA models trained on original data, orange: DCA models trained on randomized data with similar profile and phylogenetic relationships than the original data, green: independent models trained on original data) (a)** Boxplots of site-entropy values obtained with each of the three models. Boxplot center lines represent medians, box limits are upper and lower quartiles, whiskers extend to show the rest of the distribution within an 1.5× interquartile range, outliers (if any) are represented with points. Samples sizes are n=6,668 for each of the three groups. **(b)** ROC curves of different models for predicting polymorphisms observed at $> 5\%$ frequency in *E. coli*.

Supplementary References

[1] Edwin Rodriguez Horta and Martin Weigt. On the effect of phylogenetic correlations in coevolution-based contact prediction in proteins. *PLOS Computational Biology*, 17(5):e1008957, May 2021. Publisher: Public Library of Science.

[2] William P. Russ, Matteo Figliuzzi, Christian Stocker, Pierre Barrat-Charlaix, Michael Socolich, Peter Kast, Donald Hilvert, Remi Monasson, Simona Cocco, Martin Weigt, and Rama Ranganathan. An evolution-based model for designing chorismate mutase enzymes. *Science*, 369(6502):440–445, July 2020.
