# Supplementary material for: Deciphering polymorphism in 61,157 Escherichia coli genomes via epistatic sequence landscapes
Source: Nat Commun. 2022 Jul 12;13:4030. doi: 10.1038/s41467-022-31643-3 (PMC9276797; doi:10.1038/s41467-022-31643-3)
Supplement: Supplementary file 3 — Reporting Summary [file 41467_2022_31643_MOESM3_ESM.pdf]

nature portfolio

Corresponding author(s): Martin Weigt

Last updated by author(s): Dec 13, 2021

# Reporting Summary

Nature Portfolio wishes to improve the reproducibility of the work that we publish. This form provides structure for consistency and transparency in reporting. For further information on Nature Portfolio policies, see our Editorial Policies and the Editorial Policy Checklist.

## Statistics

For all statistical analyses, confirm that the following items are present in the figure legend, table legend, main text, or Methods section.

| n/a | Confirmed | |
|---|---|---|
| ☐ | ☒ | The exact sample size (*n*) for each experimental group/condition, given as a discrete number and unit of measurement |
| ☒ | ☐ | A statement on whether measurements were taken from distinct samples or whether the same sample was measured repeatedly |
| ☒ | ☐ | The statistical test(s) used AND whether they are one- or two-sided<br>*Only common tests should be described solely by name; describe more complex techniques in the Methods section.* |
| ☒ | ☐ | A description of all covariates tested |
| ☒ | ☐ | A description of any assumptions or corrections, such as tests of normality and adjustment for multiple comparisons |
| ☐ | ☒ | A full description of the statistical parameters including central tendency (e.g. means) or other basic estimates (e.g. regression coefficient) AND variation (e.g. standard deviation) or associated estimates of uncertainty (e.g. confidence intervals) |
| ☒ | ☐ | For null hypothesis testing, the test statistic (e.g. *F*, *t*, *r*) with confidence intervals, effect sizes, degrees of freedom and *P* value noted<br>*Give P values as exact values whenever suitable.* |
| ☒ | ☐ | For Bayesian analysis, information on the choice of priors and Markov chain Monte Carlo settings |
| ☒ | ☐ | For hierarchical and complex designs, identification of the appropriate level for tests and full reporting of outcomes |
| ☐ | ☒ | Estimates of effect sizes (e.g. Cohen's *d*, Pearson's *r*), indicating how they were calculated |

*Our web collection on statistics for biologists contains articles on many of the points above.*

## Software and code

Policy information about availability of computer code

| | |
|---|---|
| Data collection | HMMER 3.3.1 (for phmmer and hmmalign), HHblits 3.3 |
| Data analysis | Prokka 1.13.3, mafft v7.471, vsearch v2.15.1, FastTree 2.1.3, plmDCA (https://github.com/pagnani/PlmDCA), DCAUtils (https://github.com/carlobaldassi/DCAUtils.jl), Python3 v3.8, BioPython v1.77, custom code available at https://github.com/LucileVG/DCA_polymorphism_Ecoli and linked to Zenodo database (DOI: 10.5281/zenodo.6624449). |

For manuscripts utilizing custom algorithms or software that are central to the research but not yet described in published literature, software must be made available to editors and reviewers. We strongly encourage code deposition in a community repository (e.g. GitHub). See the Nature Portfolio guidelines for submitting code & software for further information.

## Data

Policy information about availability of data

All manuscripts must include a data availability statement. This statement should provide the following information, where applicable:

- Accession codes, unique identifiers, or web links for publicly available datasets
- A description of any restrictions on data availability
- For clinical datasets or third party data, please ensure that the statement adheres to our policy

The sequence data is available on Zenodo (DOI: 10.5281/zenodo.5774192).
The exact list of genes and Pfam domains analysed is available at https://github.com/LucileVG/DCA_polymorphism_Ecoli/gene_domains.csv.
The following public databases were used: UniRef30 (2020-03), Pfam 34.0 (March 2021), Enterobase and Mage.
The reference genomes used in this study are the following:
GA4805AA genome (available on NCBI under BioProject accession id PRJNA218163 [https://www.ncbi.nlm.nih.gov/bioproject/PRJNA218163/])

Escherichia coli K12 - chromosome ECK.1 [https://mage.genoscope.cns.fr/microscope/mage/viewer.php?O_id=149]
Escherichia coli UMN026 - chromosome ESCUM.2 [https://mage.genoscope.cns.fr/microscope/mage/viewer.php?O_id=127]
Escherichia albertii TW07627 - chromosome ESCAL.1 [https://mage.genoscope.cns.fr/microscope/mage/viewer.php?O_id=601]
Escherichia fergusonii ATCC 35469T - chromosome EFER.2 [https://mage.genoscope.cns.fr/microscope/mage/viewer.php?O_id=138]
Salmonella enterica subsp. arizonae serovar 62:z4,z23:-- RSK2980 - chromosome NC_010067.1 [https://mage.genoscope.cns.fr/microscope/mage/viewer.php?O_id=1484]
Klebsiella pneumoniae 1162281 - WGS AFQL.1 [https://mage.genoscope.cns.fr/microscope/mage/viewer.php?O_id=7390]
Atlantibacter hermannii 4928STDY7071316 - WGS CABGLB01.1 [https://mage.genoscope.cns.fr/microscope/mage/viewer.php?O_id=12477]
Pantoea ananatis AJ13355 - chromosome NC_017531.1 [https://mage.genoscope.cns.fr/microscope/mage/viewer.php?O_id=7637]
Yersinia pestis Angola - chromosome NC_010159.1 [https://mage.genoscope.cns.fr/microscope/mage/viewer.php?O_id=356]

# Field-specific reporting

Please select the one below that is the best fit for your research. If you are not sure, read the appropriate sections before making your selection.

☐ Life sciences ☐ Behavioural & social sciences ☒ Ecological, evolutionary & environmental sciences

For a reference copy of the document with all sections, see nature.com/documents/nr-reporting-summary-flat.pdf

# Ecological, evolutionary & environmental sciences study design

All studies must disclose on these points even when the disclosure is negative.

| | |
|---|---|
| Study description | The study is performed on data available in public databases (cf. Data section above). |
| Research sample | No research sample was collected. |
| Sampling strategy | No research sample was collected. |
| Data collection | No research data was collected (cf. Data section) |
| Timing and spatial scale | No research data was collected (cf. Data section) |
| Data exclusions | No research data was collected (cf. Data section) |
| Reproducibility | No research data was collected (cf. Data section) |
| Randomization | No research data was collected (cf. Data section) |
| Blinding | No research data was collected (cf. Data section) |

Did the study involve field work? ☐ Yes ☒ No

# Reporting for specific materials, systems and methods

We require information from authors about some types of materials, experimental systems and methods used in many studies. Here, indicate whether each material, system or method listed is relevant to your study. If you are not sure if a list item applies to your research, read the appropriate section before selecting a response.

## Materials & experimental systems

| n/a | Involved in the study |
|---|---|
| ☒ | ☐ Antibodies |
| ☒ | ☐ Eukaryotic cell lines |
| ☒ | ☐ Palaeontology and archaeology |
| ☒ | ☐ Animals and other organisms |
| ☒ | ☐ Human research participants |
| ☒ | ☐ Clinical data |
| ☒ | ☐ Dual use research of concern |

## Methods

| n/a | Involved in the study |
|---|---|
| ☒ | ☐ ChIP-seq |
| ☒ | ☐ Flow cytometry |
| ☒ | ☐ MRI-based neuroimaging |
